# Young Children’s Indiscriminate Helping Behavior Toward a Humanoid Robot

**DOI:** 10.3389/fpsyg.2020.00239

**Published:** 2020-02-21

**Authors:** Dorothea U. Martin, Madeline I. MacIntyre, Conrad Perry, Georgia Clift, Sonja Pedell, Jordy Kaufman

**Affiliations:** ^1^Swinburne BabyLab, Department of Psychological Sciences, Swinburne University of Technology, Hawthorn, VIC, Australia; ^2^School of Psychology, The University of Adelaide, Adelaide, SA, Australia; ^3^Swinburne Future Self and Design Living Lab, Centre for Design Innovation, Swinburne University of Technology, Hawthorn, VIC, Australia

**Keywords:** prosocial behavior, altruism, helping, animacy, social robotics, human-robot interaction, child-robot interaction

## Abstract

Young children help others in a range of situations, relatively indiscriminate of the characteristics of those they help. Recent results have suggested that young children’s helping behavior extends even to humanoid robots. However, it has been unclear how characteristics of robots would influence children’s helping behavior. Considering previous findings suggesting that certain robot features influence adults’ perception of and their behavior toward robots, the question arises of whether young children’s behavior and perception would follow the same principles. The current study investigated whether two key characteristics of a humanoid robot (animate autonomy and friendly expressiveness) would affect children’s instrumental helping behavior and their perception of the robot as an animate being. Eighty-two 3-year-old children participated in one of four experimental conditions manipulating a robot’s ostensible animate autonomy (high/low) and friendly expressiveness (friendly/neutral). Helping was assessed in an out-of-reach task and animacy ratings were assessed in a post-test interview. Results suggested that both children’s helping behavior, as well as their perception of the robot as animate, were unaffected by the robot’s characteristics. The findings indicate that young children’s helping behavior extends largely indiscriminately across two important characteristics. These results increase our understanding of the development of children’s altruistic behavior and animate-inanimate distinctions. Our findings also raise important ethical questions for the field of child-robot interaction.

## Introduction

Humans behave prosocially in a wide range of situations and this prosocial behavior begins to emerge at a very early age (e.g., see, Warneken and Tomasello, 2009; Svetlova et al., 2010; Dunfield, 2014). Infants as young as 12 months of age provide others with information by pointing to objects (Liszkowski et al., 2006, 2008), 18-months-olds show concern for others in distress and comfort them (Zahn-Waxler et al., 1992; Vaish et al., 2009), and by the end of the second year of life, they readily share resources with others (Hay et al., 1991; Brownell et al., 2009; Dunfield et al., 2011). Young children also help others achieve certain goals and this instrumental helping begins to emerge as soon as toddlers are physically able to do so (e.g., Rheingold, 1982; Warneken and Tomasello, 2006, 2007; Svetlova et al., 2010; Dunfield et al., 2011; Hepach et al., 2012). For instance, 14-months-olds reliably hand over out-of-reach objects to adults (Warneken and Tomasello, 2007) and by 18 months of age, children help in cognitively more demanding tasks such as removing obstacles (Warneken and Tomasello, 2006) and correcting an adult who is about to commit a mistake (Knudsen and Liszkowski, 2012) while taking into account false beliefs (Buttelmann et al., 2009; Knudsen and Liszkowski, 2011).

Young children not only help in a wide range of situations, they also help partners with different characteristics. They help same-aged peers (Hepach et al., 2017), familiar (e.g., Warneken and Tomasello, 2006, 2007; Allen et al., 2018) and unfamiliar adults (Rheingold, 1982; Hepach et al., 2016), and even recipients who had behaved antisocially (Dahl et al., 2013; Sebastián-Enesco et al., 2013; c.f., Vaish et al., 2010). Furthermore, a recent study showed that young children’s helping behavior is not confined to human recipients, but extends to a robot in need (Martin et al., 2020; for a study with older children, see Beran et al., 2011). In particular, using a procedure based on research by Warneken and Tomasello (2006, 2007), Martin et al. (2020) presented 3-year-old children with a humanoid robot that played a xylophone and subsequently dropped the xylophone stick out of its reach. Children were likely to help the robot by returning the stick when it appeared to need help (i.e., when it dropped the stick seemingly accidentally and reached for it). In contrast, children who were presented with the same situation in which the robot did not indicate a need for help (i.e., when it dropped the stick seemingly intentionally and did not reach for it) were far less likely and slower to help. These results suggest that young children attribute goals to a humanoid robot and are motivated to help it (Martin et al., 2020). These findings are consistent with previous results on children’s instrumental helping behavior (e.g., Warneken and Tomasello, 2006, 2007), indicating that young children’s helping behavior extends almost indiscriminately across recipients with varying characteristics (e.g., Hay, 1994; Warneken and Tomasello, 2009).

Given the lack of previous research investigating young children’s instrumental helping behavior toward robots, the study by Martin et al. (2020) was designed to allow for comparisons with previous studies using human recipients (e.g.,Warneken and Tomasello, 2006, 2007). Thus, the pre-programmed robot followed a pre-determined behavioral and verbal script. The script incorporated several features that were designed to maintain similarity with previous studies using human recipients. However, several studies have shown that people’s perception of and behavior toward robots depend on robots’ physical and behavioral cues (e.g., Bartneck et al., 2007; Somanader et al., 2011; Srinivasan and Takayama, 2016).

One particularly important feature is the robots’ apparent autonomy – a feature that was incorporated and unvaried in the study by Martin et al. (2020). The importance of autonomy for adults’ prosocial behavior was highlighted in a study by [Srinivasan and Takayama (2016), Experiment 2]. They showed that adults who were presented with a cleaning robot that behaved seemingly autonomously were significantly faster to comply with a request for help made by the robot than participants who were in the same situation but believed the robot to be tele-operated. Moreover, although children report similar enjoyment in interactions with humanoid robots that they believe to be tele-operated or autonomous, they attribute lower intelligence to the tele-operated robot (Tozadore et al., 2017). These findings suggest that seemingly autonomously behaving robots might be perceived as more animate than tele-operated robots. In contrast, perceived low autonomy might facilitate perceptions of robots as machine-like (Kahn et al., 2007).

Supporting this idea are studies showing that both adults (Fukuda and Ueda, 2010) and children (Somanader et al., 2011) attribute more animacy (i.e., lifelikeness; properties of living beings) to a robot that moves autonomously and in a goal-directed way than to a robot that is tele-operated. Horstmann et al. (2018) showed that this effect of autonomy may not be restricted to the robot’s movements, but may also apply to its verbal behavior. In this study, Horstmann et al. (2018) investigated adults’ willingness to switch off a humanoid robot that was either programmed to behave humanlike (e.g., it disclosed personal preferences and used humor) or machinelike (e.g., it appeared functional and non-personal). When given a choice to turn off the robot after an interaction, adults in both conditions were equally likely to do so, however, participants were less likely to switch it off when the robot’s raised objections against being switched off. Interestingly, however, participants who interacted with a machinelike robot that raised objections hesitated the most. As argued by Horstmann et al. (2018), it is possible that the latter result stems from participants’ cognitive conflict regarding why a previously machinelike robot would suddenly act more autonomous. Furthermore, the results by Horstmann et al. (2018) showed that adults perceived the machinelike robot as less likeable than the humanlike robot. Taken together with results showing that adults empathize more with humanlike compared to machinelike robots (Riek et al., 2009), these findings highlight the effects of perceived autonomy on people’s perception of and behavior toward robots.

As indicated by the findings of Horstmann et al. (2018), another important aspect that could affect people’s behavior toward robots concerns the robots’ social skills. Further support for this claim stems from a study by Bartneck et al. (2007), in which adult participants were instructed to play a game with an iCat robot that has a humanoid face and can mimic human facial expressions. Bartneck et al. (2007) varied both the robot’s intelligence (high vs. low) and agreeableness (high vs. low). When the game ended, the experimenter asked each participant to turn off the robot by turning a switch, which ostensibly would erase the robot’s memory and personality. Immediately after participants had received these instructions, the robot begged to remain switched on. Results showed that both intelligence and agreeableness of the robot affected participants’ willingness to switch it off; participants hesitated significantly longer to switch off the robot when it appeared highly intelligent or agreeable.

While Bartneck et al. (2007) varied an ostensible personality trait of the robot, other studies have focused more on the effects of robots’ emotional expression. For instance, Złotowski et al. (2014) found that adults rated the animacy of a humanoid robot that expressed positive and negative emotions higher than that of a robot, which reacted unemotionally. Similarly, 8- and 9-year-old-children were found to show more positive expressions (e.g., smiles, positive verbalizations) and fewer negative expressions (e.g., frowns, negative vocalizations) toward an affective robot, which expressed emotions using its voice and gestures, compared to a non-affective robot, which showed random expressions (Tielman et al., 2014). As suggested by Niculescu et al. (2013) robots’ voice-pitch variation might be a further aspect that could affect people’s perceived emotionality of robots. Specifically, Niculescu et al. (2013) found that adults rated the overall interaction with a robot as well as its overall appeal as more enjoyable when the robot’s voice exhibited a relatively high pitch variation (i.e., with high emotional expression) than when it had a relatively low pitch variation (i.e., monotonous).

Overall, these findings suggest potential effects of robots’ characteristics, such as its perceived autonomy, animacy, and emotional expressiveness, on human behavior toward robots. Thus, it is possible that these robot characteristics affected children’s helping behavior toward the robot in the study by Martin et al. (2020). In particular, the robot in the study by Martin et al. (2020) showed a high degree of autonomy (i.e., children were oblivious that the robot was tele-operated by a second experimenter in an adjacent room) and expressed its own preferences. Furthermore, the robot’s voice had a high pitch variation, sounding friendly and expressive. It is possible that these robot features have contributed to both children’s helping behavior as well as to their perception of the robot as an animate being. This possibility would align with some previous results of studies with older children and adults (e.g., Somanader et al., 2011; Niculescu et al., 2013; Złotowski et al., 2014; Horstmann et al., 2018). In contrast, other researchers have argued that young children are rather indiscriminate in their prosocial behavior (Hay, 1994; Hay and Cooke, 2007; Warneken and Tomasello, 2009; Tomasello, 2014), indicating that the robot features mentioned above might have little or no effect. Indeed, some studies have suggested that children’s helping behavior may be unaffected by characteristics of the recipient, such as age (e.g., Hepach et al., 2017), familiarity (e.g., Hepach et al., 2016), and previous behavior (e.g., Sebastián-Enesco et al., 2013). However, the scope of previously explored factors is relatively narrow, allowing further exploration of what may affect young children’s willingness to help.

The current experiment was designed to extend previous investigations on the indiscriminate nature of young children’s helping behavior and apply them to a novel recipient; a robot. Although there are many robot characteristics that could potentially affect human behavior and perception, previous research has indicated that a robot’s animacy, including autonomy, as well as its expressiveness are of particular importance (e.g., Somanader et al., 2011; Niculescu et al., 2013; Srinivasan and Takayama, 2016). Thus, although the results by Martin et al. (2020) indicate that young children’s helping behavior extends to a robot, children’s willingness to help may have been increased by certain features of the robot such as the robot’s seemingly autonomous behavior and preferences as well as its friendly expressiveness. To explore this possibility, the current study investigated whether a robot’s animate autonomy and expressiveness would affect young children’s helpful behavior toward it as well as their perceptions of the robot as animate.

To address this question, children were tested in one of four conditions that varied animate autonomy (high vs. low) and friendly expressiveness of voice (friendly vs. neutral). A high level of animate autonomy was operationalized using seemingly independent behavior, including spontaneous movements and verbal behavior, as well as verbal statements expressing its own thoughts and preferences. In contrast, under conditions of low animate autonomy, all verbal and non-verbal behavior depended on visible human operation and the robot did not express any thoughts and preferences. The expressiveness of the robot’s voice was varied by either using a high pitch variation with an upward inflection (i.e., friendly) or a low pitch variation with a monotone inflection (i.e., neutral). The procedure used in the current study was based on the study by Martin et al. (2020). Following a warm-up phase with a humanoid robot, we presented 3-year old children with a situation in which the robot dropped an object (i.e., a xylophone stick) and reached for it unsuccessfully. Children’s helping behavior and latency to help were assessed. The experiment was followed by a post-test interview to assess three aspects of children’s perceived animacy of the robot (cognitive, affective, and physiological characteristics).

Based on previous findings, we aimed to investigate whether preschool-aged children’s helping rates and animacy perceptions would be influenced by certain robot characteristics. Given previous results showing that robots’ ostensible animacy, autonomy and social skills, including its emotional expressiveness, can foster adults’ and older children’s social behavior toward robots and increase perceptions of robots as animate (e.g., Somanader et al., 2011; Niculescu et al., 2013; Horstmann et al., 2018), it could be expected that high levels of animate autonomy and friendly expressiveness would elicit higher helping rates and animacy perceptions than low levels of animate autonomy and friendly expressiveness. In contrast, another line of previous research suggests that young children’s helping behavior is largely unaffected by aspects of the recipient (e.g., Sebastián-Enesco et al., 2013; Hepach et al., 2016, 2017; also see, Hay, 1994; Warneken and Tomasello, 2009). By the latter account, children’s helping behavior and perception of animacy toward a robot would be unaffected by variations of animate autonomy and friendly expressiveness (also see Okita and Schwartz, 2006).

## Materials and Methods

### Participants

Data was collected over a period of 14 months. A total of 82 participants (each *n* = 21 in both low animate autonomy conditions; each *n* = 20 in both high animate autonomy conditions) contributed data to the study. Participants (34 females, 48 males) were typically developing children between the ages of 36 and 47 months (*M* = 41.30, *SD* = 3.27). An additional 16 children were excluded due to fussiness (*n* = 11), procedural error (*n* = 3), and robot malfunctioning (*n* = 2). Participants were recruited from surrounding suburbs in the university’s greater metropolitan area. Parents’ median reported household income was in the A$100,000 to A$150,000 range. Parents most commonly identified as Anglo-Australian (60%), mixed ethnicity (12%), Asian/Indian (9%), and English (7%). The majority of children (95%) spoke English as a first language.

The study was approved by the host university’s human research ethics committee. Each caregiver provided informed consent for their child’s participation. Caregivers supplied information regarding their child’s previous experience with live robots. Eighteen percent of caregivers (*n* = 15) reported that their child owned a toy robot.

### Materials

The robot used in this study was a programmable humanoid NAO robot (Aldebaran Robotics). The robot stands at 58 cm tall, is fitted with two speakers and four microphones, and is equipped with a “life mode” setting. When set to “life mode”, the robot turns its head toward the loudest source of sound. All vocalizations of the robot were pre-recorded by an adult human female voice actor (for examples of verbal statements see Table 1). Two versions of recordings were used; a highly friendly version with an upward inflection (the same recordings used as in Martin et al., 2020) and a neutral version with a monotone inflection (for examples see Supplementary Material).

**TABLE 1:** Excerpts of the warm-up phase scripts.

**High Autonomy**	**Low Autonomy**
E1: “Would you like to ask the robot what its name is?”	E1: “This robot has a number. Press this button and it will tell us.”
Robot: “Hello! My name is Kira. Welcome to the Babylab!”	Robot: “My ID number is 19469233” E1: “Press the button and the robot will ask you a question.”
Robot: “What’s your name?”	Robot: “What’s your name?”
Robot: “Nice to meet you.”	
E1: “Would you like to ask Kira what her favorite color is?”	E1: “This robot can say different colors. Do you want to press the button? It will tell us what color this is.” (E1 holds up a blue sheet).
Robot: “My favorite color is blue.”	Robot: “Blue.”
	E1: “Press the button and the robot will ask another question.”
Robot: “What is your favorite color?”	Robot: “What is your favorite color?”
E1: “Would you like to ask Kira what her favorite food is?”	E1: “When you press the button, the robot will tell us what it eats.”
Robot: “My favorite food is ice cream.”	Robot: “I don’t eat food, I get my energy by being plugged into the wall.”
	E1: “The robot will ask you a question when you press the button.”
Robot: “What is your favorite food?”	Robot: “What is your favorite food?”
E1: “I think Kira likes music.”	E1: “The robot can play music. If you press the button it will ask a question.”
Robot: “I love music!”	
Robot: “Do you like music?”	Robot: “Do you like music?”
Robot: “This is my favorite song: (sings for 30 s).”	Robot: “This is my favorite song: (sings for 30 s).”

The test room was fitted with two cameras, a microphone, a round table (height: 43 cm, diameter: 60 cm), a rattle, and a xylophone. A control room, adjacent to the test room, was equipped with a laptop including Choregraphe software (Aldebaran Robotics), a monitor displaying a live video feed of the test room, and headphones providing a live audio feed of the test room.

Experimenter 2 (E2) controlled the NAO wirelessly from the control room. Four distinct, pre-programmed scripts were used, one for each condition. Following the script, E2 chose to initiate the appropriate verbal responses and pre-selected movements, which were then executed by the robot.

**FIGURE 1:**
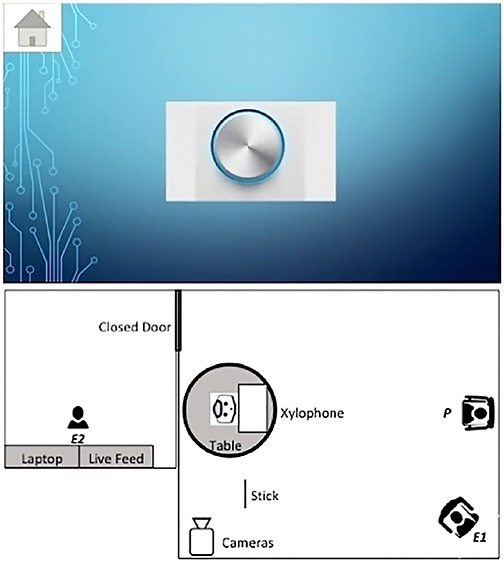
Robot control program as displayed on the tablet **(top)**; test room set-up during the test phase **(bottom)**.

Children in the low animate autonomy conditions were provided with a tablet device. Children were informed that they could control the robot using a *Robot Control Program* on the tablet device, which in fact was a PowerPoint presentation slide with an illustration of a button (see Figure 1). Touching the button resulted in a clicking sound and a simulated inward movement of the button.

### Procedure and Design

The experiment consisted of four phases; a free play phase (10 min), warm-up phase (7 min), test phase (2 min), and post-test animacy interview (10 min). Prior to the child’s arrival to the lab, each child was randomly assigned to one of four conditions, varying the degree of the robot’s animate autonomy (high vs. low) and friendly expressiveness (friendly vs. neutral) during the warm-up phase, resulting in the conditions: High Animate Autonomy/Friendly (HAAF), High Animate Autonomy/Neutral (HAAN), Low Animate Autonomy/Friendly (LAAF), Low Animate Autonomy/Neutral (LAAN). The procedures of the remaining three phases remained unvaried across all conditions. The role of experimenter 1 (E1) was shared between three female adults.

#### Free Play Phase

Each session began with a free play phase, in which E1 familiarized the child with the lab environment and obtained informed consent from the caregiver. Subsequently, the caregiver was asked to leave the room while E1 and the participant remained in the test room. Experimenter 2 (E2) then carried the robot into the test room. E2 left the test room to surreptitiously control the robot from an adjacent room. Subsequently, each participant was familiarized with the robot in a warm-up phase.

#### Warm-Up Phase

The warm-up phase followed a predefined script during which the robot was set to life mode. The script (for examples see Table 1; for full scripts see Supplementary Material) determined the responses of E1 and the robot. The robot’s animate autonomy (high vs. low) and friendly expressiveness (friendly vs. neutral) were varied in four between-subjects conditions.

##### Friendly expressiveness

In order to vary friendly expressiveness between the friendly and neutral conditions, the robot’s voice either appeared friendly with an upward inflection and a high pitch variation (in HAAF and LAAF) or neutral with a monotone inflection and a low pitch variation (in HAAN and LAAN).

##### Animate autonomy

The high animate autonomy conditions followed the script used by Martin et al. (2020). Specifically, the robot appeared to behave independently, showing seemingly spontaneous verbal and non-verbal behaviors (see Table 1; for full scripts see Supplementary Material). In addition to the behaviors in the pre-determined script, E2 could select from a number of six spontaneous exclamations in response to the child (e.g., “Nice!”, “Interesting!”).

The warm-up phase began with the robot introducing itself as *Kira*. E1 encouraged the child to engage in conversation with the robot about certain topics, such as favorite foods and favorite colors. Subsequently, E1 encouraged the child to play a game, in which the robot played three to five animal sounds and the child had the opportunity to guess the corresponding animal. Following the game, the robot expressed interest in music. The robot sang a song, played a rattle, and made statements about playing the xylophone. At this point, E2 turned off the robot’s life mode so that the robot would not turn its head to the loudest source of sound. E1 then placed the robot and the xylophone on the table, put a stick into the robot’s hand, and asked the child to watch the robot. E1 turned away from the child and robot to ostensibly read some papers. At this point, the test phase began.

In the low animate autonomy conditions, children were provided with a tablet device at the beginning of the warm-up phase. E1 explained that they could control the robot by pressing the button in the robot control program. As in the high animate autonomy conditions, E2 controlled the robot’s responses from the adjacent room. Using the video and audio live feed, E2 waited until the child had pressed the button before initiating the robot’s actions.

The procedure used in the low animate autonomy conditions included the same topics as the procedure used in the high animate autonomy conditions. However, instead of exhibiting seemingly spontaneous verbal and non-verbal behaviors, the robot only responded once the button in the robot control program was pressed. Throughout the warm-up phase, E1 encouraged the child to press the button in the robot control program. If the child did not press the button despite encouragement, E1 pressed it.

Further alterations concerned verbal expressions of its animate autonomy. For instance, the robot stated an ID number rather than a name and it could name a color rather than stating its favorite color (for further comparisons see Table 1; for full scripts of both high and low animate autonomy conditions see Supplementary Material). The robot did not make any spontaneous exclamations in response to the child’s statements.

At the end of the warm-up phase, the robot’s life mode was turned off. E1 lifted the robot onto the table and pressed the button to make the robot take the xylophone stick. E1 then pointed out that she would press the button to make the robot play the xylophone. Subsequently, E1 clearly stated that she would put the tablet out of reach and that she would not be able to control the robot. Thus, during the test phase neither E1 nor the participant was holding the tablet. This was to rule out two alternative explanations for children’s motivation to return the stick. First, if E1 had hold of the tablet, children might be motivated to help E1 and this help could be mediated by returning the stick to the robot. Second, if the child had hold of the tablet, children might return the stick so they could continue to control the robot sooner. Thus, E1 pressed the button to make the robot play the xylophone and subsequently placed the tablet on a high shelf, asked the child to watch the robot, and turned away from the child and robot to ostensibly read some papers.

#### Test Phase

The test phase was based on the procedure used in Martin et al. (2020). After the robot had played the xylophone for 10 s, it dropped the stick on the floor (see Figure 1). Children’s behavior was assessed for the 30-s period after the stick was dropped.

During the first 10 s of the trial, the robot appeared to look at the stick and reach for it unsuccessfully. Subsequently, for a duration of 10 s, it alternated gaze between the stick and the approximate position of the child (i.e., the child’s chair) while reaching for the stick. The robot then exclaimed “My stick!” and continued reaching and alternating gaze for 10 s.

Any attempt to hand the stick to the robot was interpreted as helping. E1 assisted children if they clearly attempted but failed to put the stick into the robot’s hand. In the case of high animate autonomy conditions E1 directly assisted the child with putting the stick into the robot’s hand. When children in low animate autonomy conditions helped, E1 took the tablet device off the shelf and pressed the button while stating that pressing the button is necessary for the robot to grasp the stick. If the participant did not attempt to help, E1 handed the stick to the robot after the trial had ended; either directly (in high animate autonomy conditions) or after E1 had pressed the button (in low animate autonomy conditions). After the robot had received the stick, it resumed playing for 5 s (either seemingly autonomously or after E1 had pressed the button). Subsequently, E1 carried the robot into an adjacent room and returned to the test room, shut the door, and began the animacy interview.

#### Animacy Interview

E1 and the participant sat down at the table. In the 10-minute interview, based on one described by Lillard et al. (2000) and used by Martin et al. (2020), children were presented with a set of eight pictures. Two pictures each showed one photograph out of four categories (two living and two non-living categories): children (male, female), animals (cat, rabbit), robots (familiar robot, unfamiliar robot), and vehicles (car, motorbike). Pictures were presented one at the time. To each participant, the set of pictures was presented three times in an identical order. Each presentation of the complete set was paired with one of three questions. The question was repeated for each picture.

Each participant was asked a total of three questions assessing the perceived cognitive, affective, and physiological characteristics of each entity. The questions used were: “Can [item] think?”, “If everyone left and nobody is around would [item] feel lonely?”, “Can [item] breathe?”. At the beginning of the first presentation of each picture, children were also asked to state what was shown in each picture. Picture presentation and question order were counterbalanced across participants.

#### Video Coding

The coding scheme followed the scheme used by Martin et al. (2020). Helping behavior was coded as a dichotomous variable. For children who helped, latency to help was also coded. Latency to help was determined by subtracting trial onset time (stick hitting the ground) from helping time. For helping behavior, a second rater coded 37% (*n* = 30) of the videos. Inter-rater reliability was perfect with 100% agreement. Inter-rater reliability for latency of help was based on 53% (*n* = 21) of the videos of children that helped, and showed nearly perfect agreement, *r* = 0.98, *n* = 21, *p* < 0.001. Children’s answers in the interview were coded by assistants blind to the assigned condition. Children’s responses to each interview question were coded as 1 (yes) or 0 (no). An animacy score was calculated for each item presented in the interview. This score was computed as the average of the *breathe*, *feel*, and *think* scores. The score ranged from 0 (answered all three questions about an item with “no”) to 1 (answered all three questions about an item with “yes”).

## Results

### Helping

**FIGURE 2:**
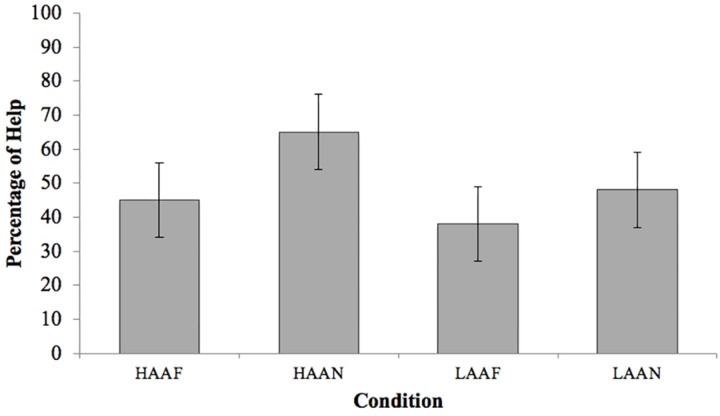
Percentage of children helping by condition. Error bars represent standard errors. Help was provided by *n* = 9 in HAAF (45%), *n* = 13 in HAAN (65%), *n* = 8 in LAAF (38%), and *n* = 10 in LAAN (48%).

Our analyses were primarily aimed at exploring how animate autonomy and friendly expressiveness affected children’s likelihood to help the robot. Help was provided by *n* = 9 in HAAF, *n* = 13 in HAAN, *n* = 8 in LAAF, and *n* = 10 in LAAN. The percentages of children providing help in each condition are reported in Figure 2.

Preliminary analyses revealed no significant main effects or interactions involving age or experimenter. Subsequently, age and experimenter were removed from the main analysis. Ownership of toy robots was too uncommon to address statistically (*n* = 15). Additional preliminary analyses identified sex as impacting the tendency to help. Sex was therefore a categorical predictor variable in the main analysis described below.

A binary logistic regression on helping behavior was conducted with the between-subjects factors animate autonomy, friendly expressiveness, and sex. There was a significant effect of sex, *χ*^2^ (1) = 5.86, *p* = 0.016, reflecting that help was offered more by male (60%) than female (32%) participants.

The analysis did not reveal any significant main effects of animate autonomy, *χ*^2^ (1) = 1.04, *p* = 0.31, or friendly expressiveness, *χ*^2^ (1) = 1.33, *p* = 0.25. There were also no significant interactions involving animate autonomy, friendly expressiveness, and sex (all *p* > 0.15).

For children who helped, the latency of helping behavior was analyzed. In all conditions, children helped after a brief delay (*M* = 7.12 s, *SD* = 4.22, also see Table 2). A two-way ANOVA revealed no significant effects of animate autonomy, *F*(1,36) < 0.01, *p* = 0.98, or of friendly expressiveness on latency to help, *F*(1,36) = 0.32, *p* = 0.58, or any significant interactions, *F*(1,36) = 0.01, *p* = 0.91.

**TABLE 2:** Means and standard deviations of latency to help in all conditions.

	***n***	**Mean (in sec)**	**Standard deviation**
HAAF	9	7.65	5.97
HAAN	13	6.69	3.29
LAAF	8	7.51	3.91
LAAN	10	6.88	4.27

### Animacy Interview

Data from participants answering less than 70% of items were excluded from this analysis (*n* = 4). Preliminary analyses revealed no main effects or interactions involving sex and age. Subsequently, age and sex were removed as factors from the main analysis. Ownership of toy robots was too uncommon to address statistically (*n* = 15).

**FIGURE 3:**
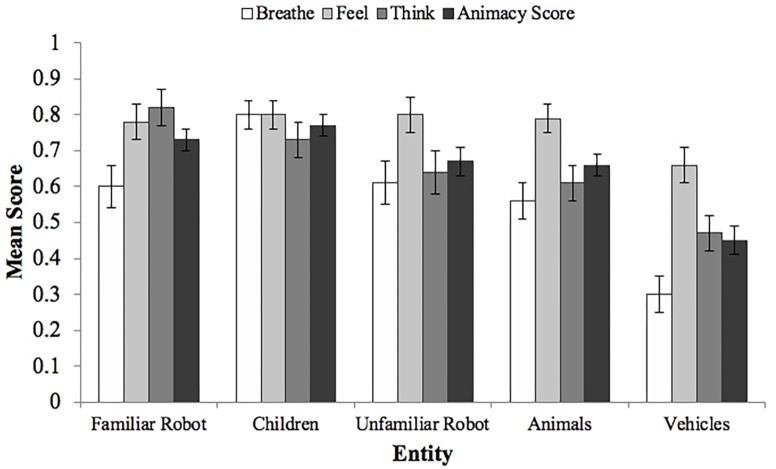
Mean scores and standard errors for each question by entity. Animacy scores are the means of the breathe, feel, and think scores. The animacy score of vehicles was significantly lower than the animacy scores of all other entities (each *p* < 0.001). The animacy score of children was significantly greater than the scores of animals, *p* = 0.01, and of the unfamiliar robot, *p* = 0.038.

The mean scores for all entities are presented in Figure 3. A within-subjects ANOVA on animacy scores revealed a significant effect of entity, *F*(4,296) = 25.47, *p* < 0.0001. A *post hoc* Tukey test showed that the score of vehicles was significantly lower than the scores of children, animals, the familiar robot, and the unfamiliar robot (each *p* < 0.001). The animacy score of children was significantly greater than the scores of animals, *p* = 0.01, and of the unfamiliar robot, *p* = 0.038.

An ANOVA revealed no significant main effects of animate autonomy, *F*(3,74) = 0.10, *p* = 0.75, or of friendly expressiveness, *F*(3,74) = 0.43, *p* = 0.52, or any significant interaction, *F*(3,74) = 1.64, *p* = 0.21, on the perceived animacy of the robot. Further analyses did not reveal any significant main effects of animate autonomy, or of friendly expressiveness or any significant animate autonomy × friendly expressiveness interactions on the perceived animacy of the remaining four entities.

We also analyzed whether there was a statistical relationship between helping and animacy scores. Analyses did not reveal any significant relationship between helping and animacy scores of all entities, *F*(1,76) = 0.09, *p* = 0.77, or any significant interactions involving helping and animacy scores, *F*(4,73) = 1.23, *p* = 0.31.

### Comparison With Martin et al. (2020)

On the grounds that the HAAF condition followed the same procedure as the experimental condition used in Martin et al. (2020), further analyses were performed to examine differences between these two studies. Although children’s helping rates were lower in the HAAF condition of the current study (70% in Martin et al., 2020, vs. 45% in the current study), an ANOVA revealed the difference to be not significant, *F*(1,38) = 2.60, *p* = 0.12 (also see Supplementary Material for a comparison of descriptive statistics).

Furthermore, although the animacy ratings followed the same pattern as in Martin et al. (2020), the animacy ratings of all item categories were lower in the current study. An ANOVA revealed a significant main effect of experiment on the overall animacy rating of entities across all conditions used in both studies, *F*(1,110) = 5.98, *p* = 0.016, but no significant experiment x entity interactions, *F*(4,440) = 0.60, *p* = 0.66. A further ANOVA was conducted to assess differences between the HAAF condition used in the current study and the experimental condition used by Martin et al. (2020). Results revealed significant main effects of experiment on the animacy rating of entities, *F*(1,35) = 4.23, *p* < 0.047, and of entity on animacy rating, *F*(4,32) = 9.07, *p* < 0.0001, but no significant experiment x entity interactions, *F*(4,32) = 0.25, *p* < 0.91.

The difference between the current results and the results by Martin et al., 2020) could not be explained by differences in the materials and procedure (i.e., the same rooms, materials and pre-programmed robot script as in Martin et al. (2020) were used in the current study). Furthermore, although in the current experiment, the role of E1 was shared between three experimenters, whereas the study by Martin et al. (2020) was conducted by one experimenter, preliminary analyses did not reveal significant experimenter effects on helping nor animacy perceptions. Participants in both studies shared a similar demographic background. Although robot ownership was more likely in the current study than in Martin et al. (2020), preliminary analyses revealed no significant differences in children’s robot ownership between the total samples of both studies.

## Discussion

The current experiment investigated whether animate autonomy and friendly expressiveness of a humanoid robot would affect 3-year-old children’s instrumental helpful behavior toward it. Results showed that children helped at a similar rate and after a similar delay in all four conditions. Thus, children’s helping behavior appeared unaffected by the robot’s level of animate autonomy and friendly expressiveness of voice. Furthermore, interview results suggested that children’s perception of the robot as animate did not differ as function of the robot’s animate autonomy or friendly expressiveness. These findings may have important implications for psychological theories of young children’s helping behavior, theories regarding children’s animate-inanimate distinctions, as well as for the field of child-robot interaction.

One potential explanation for these results is based on recent findings in developmental psychology showing that young children’s helping behavior is relatively robust against characteristics of human recipients in need (e.g., Warneken and Tomasello, 2007; Dahl et al., 2013; Sebastián-Enesco et al., 2013; Hepach et al., 2017). It has been proposed that young children are relatively indiscriminate in whom they help (Hay, 1994; Warneken and Tomasello, 2009). With age, children begin to discriminate more in their prosocial behavior based on the recipient’s group membership (e.g., Fehr et al., 2008; Gummerum et al., 2009; Dunham et al., 2011; Lu and Chang, 2016) and previous prosocial behavior (e.g., Warneken and Tomasello, 2013; Sebastián-Enesco and Warneken, 2015; also see Kenward and Dahl, 2011; Martin and Olson, 2015). Thus, it is possible that young children’s helping behavior extends to a robot, indiscriminately of its level of animate autonomy and friendly expressiveness.

Nevertheless, we acknowledge that it is hypothetically possible that 3-year-old children are unable to distinguish high from low levels of both animate autonomy and friendly expressiveness. While possible, it is notable that previous research has indicated that children of this age are indeed capable of discerning subtle aspects of autonomy (Meltzoff et al., 2010; Breazeal et al., 2016) and voice (Singh et al., 2002; Volkova et al., 2006; Tsang and Conrad, 2010). Thus, in regards to children’s behavior toward the robot in the current study, it is likely that the robot’s apparent need for help outweighed the level of animate autonomy and friendly expressiveness.

Another alternative explanation for why children’s helping was unaffected by low animate autonomy of the robot stems from the conditions’ potential for cognitive conflict. In particular, under conditions of low animate autonomy, children could seemingly control the robot’s actions by pressing a button in a program on a tablet device. However, this was not the case in the test phase. Specifically, once the experimenter pressed the button for the robot to play the xylophone, the experimenter put away the tablet, stating that she and the child would not need to control the robot while the robot was playing the xylophone. Subsequently, after the robot had played the xylophone for 10 s, the robot exhibited what could be interpreted as autonomous behavior: when dropping the stick, the robot exclaimed “Oh!” and reached for the stick. Subsequently, the robot alternated gaze between the stick and the child and exclaimed “My stick!”.

Although the test phase was designed to follow the same procedure in all four conditions in order to ensure comparability, the seemingly autonomous behavior in the test phase could have led to surprise and cognitive conflict in children who had previously experienced the robot as a non-autonomous agent. Supporting this explanation are previous findings by Horstmann et al. (2018), who showed that adults who had interacted with a machinelike robot hesitated to turn it off when the robot raised objections. Following this explanation, it is possible that the sudden appearance of seemingly autonomous behavior during the 30 s interval in the test phase of the current study was sufficient to elicit helping behavior. Despite its brief duration, this autonomous behavior may have operated as a strong cue, which possibly, negated children’s previous perception of the robot as inanimate. In line with this explanation, our interview data shows that children were likely to perceive the robot as animate regardless of its experimentally assigned level of animate autonomy. However, as the interview was conducted at the end of the session, it could not assess potential changes in children’s perceptions that may have been elicited by unpredicted autonomous behavior.

There is some evidence that the predictability of motion plays an important role in human perception of robot animacy. For instance, in a study by Fukuda and Ueda (2010), adults either observed or controlled a robot, which either exhibited a high degree of goal-directedness in its movements or a combination of goal-directed and random movements. When observing the robot, participants rated the robot’s animacy higher when it showed high rather than reduced levels of goal-directedness. Interestingly, the opposite result was found when participants controlled the robot; participants rated its animacy higher when it showed reduced rather than high degrees of goal-directed movements (Fukuda and Ueda, 2010). Thus, when extending these findings to the children in our current study, it is possible that moderately unpredictable behaviors of the robot under conditions of low animate autonomy (i.e., when children ostensibly controlled the robot) enforced children’s perception of the robot as animate. In line with this explanation, prior studies with children has shown that 4- to 6-year-olds are more likely to perceive a robot as animate when it acts seemingly autonomous than when it is visibly controlled (Somanader et al., 2011; Cameron et al., 2017).

It is also possible that children may have already perceived the robot as animate even before the test phase. This possibility is supported by studies showing that 3-year-old children are likely to attribute animacy to robots and often do so regardless of certain robot characteristics (e.g., Kahn et al., 2006; Okita and Schwartz, 2006; Saylor et al., 2010). For instance, Okita and Schwartz (2006) showed that 3-year-olds perceived robotic animals as highly animate, regardless of different types of robot behaviors (e.g., dancing, standing still) or contingency of its behaviors.

Another robot characteristic that was varied in the current study was the expressiveness of the robot’s voice. Although the voice was recorded from the same human voice actress, the expressiveness of the voice was either characterized by a high pitch variation and upward inflection, making it sound friendly and approachable; or by a low pitch variation and monotone inflection, making it sound neutral and machine-like. As for animate autonomy, the robot’s voice did not appear to influence children’s helping behavior, nor children’s perception of the robot as animate. The fact that expressiveness seemed to have no influence on children is interesting in the context of previous findings on how robots’ emotional expressiveness might affect human perception and behavior. For instance, Niculescu et al. (2013) found that robots’ vocal expressiveness increased adult’s rating of its likeability. Similarly, findings by Tielman et al. (2014) suggested that 8- and 9-year-old children show more positive and less negative expressions toward an emotionally expressive than a non-affective robot. Although likeability was not directly assessed in the current study, the expressiveness of voice might also operate as a cue to the robot’s social skills and in turn, affect animacy perceptions. This idea is supported by studies showing that adults perceive robots that exhibit human-like social skills as more animate (Złotowski et al., 2014) and hesitate more to switch them off than robots that lack social skills (Bartneck et al., 2007; Horstmann et al., 2018). In contrast, the current results indicate that in comparison to older children and adults, young children’s perception of and behavior toward robots may not follow the same principles.

Thus, a potential explanation for why children’s helping behavior and animacy perception in the current study appeared unaffected by the robot’s animate autonomy and expressiveness builds on children’s developing concept for animacy. Although there is debate about how this concept develops in children (e.g., Piaget, 1929; Carey, 1985; diSessa, 1988; Keil, 1989; Gopnik and Meltzoff, 1997), animate-inanimate distinctions might rely on several cues (Gelman and Spelke, 1981; Poulin-Dubois et al., 1996; Rakison and Poulin-Dubois, 2001; Opfer and Gelman, 2010). Featural cues, such as the presence of a face (Goren et al., 1975; Morton and Johnson, 1991; Johnson et al., 1998; Balas and Tonsager, 2014; also see Nelson, 2001) and eyes (Batki et al., 2000; Looser and Wheatley, 2010) might be one type of cue that is taken into account when making this distinction. Another type of cue concerns motion. In this regard, previous research has highlighted the importance of recognizing object-directed action – an ability that begins to develop in infancy (Luo and Baillargeon, 2005; Sommerville et al., 2005; Tomasello et al., 2005; Csibra, 2008; Luo, 2011; Kaduk et al., 2013). However, as infants attribute object-directed movements not only to animate but also to inanimate agents under certain circumstances, the roles of movement, particularly of the agent’s self-propulsion (Premack, 1990; Mandler, 1992, 2000; Spelke et al., 1995; Markson and Spelke, 2006), contingency and variability of its behavior (Rochat et al., 1997; Johnson et al., 1998; Gergely, 2001; Csibra, 2008) have been debated.

Although pre-school-aged children’s animate-inanimate distinctions have been shown to be of high accuracy for other entities (e.g., Gelman et al., 1983; Inagaki and Hatano, 2002, 2006; Opfer and Gelman, 2010), robots might represent a particularly difficult case as their features cross animate and machine-like cues (Saylor et al., 2010). In line with this, previous research has shown that 3-year-olds broadly attribute animacy to robots, relatively unaffected by the its characteristics (i.e., realistic appearance, responsiveness; see Okita and Schwartz, 2006), however, pre-schoolers’ ability to classify robots as inanimate becomes more accurate at around 4 years of age (Mikropoulos et al., 2003; Okita and Schwartz, 2006; Jipson and Gelman, 2007; Saylor et al., 2010; Cameron et al., 2017).

Thus, it is possible that the 3-year-old children in the current study perceived the robot as animate regardless of its animate autonomy and expressiveness. It is likely that human perception of animacy does not depend on a few specific cues but on a complex composite (Opfer and Gelman, 2010). Thus, even when animate autonomy and friendly expressiveness were reduced, features such as the robot’s face and eyes, as well as its relatively high self-propulsion (i.e., although it was visibly remote-controlled, the robot did not have to be physically moved) and a combination of contingent and non-contingent actions (i.e., in the warm-up vs. test phase) may have operated as cues to animacy and outweighed cues that indicated a lack thereof. Future studies are needed to further explore which cues primarily drive children’s animacy attributions to robots and whether the same cues would affect children’s helping behavior. One interesting avenue for future research to consider concerns the question whether children’s perception of a non-autonomous robot would change after observing brief periods of autonomous action.

Another question that warrants further investigation concerns children’s willingness to help a robot when help does not appear needed. Surprisingly, helping rates in the HAAF condition in the current study were lower than in a previous study by Martin et al. (2020), despite the same procedure being used. Although this difference was not significant, it leaves open the question whether children in the current study were less willing to help a robot in need or simply less willing to engage with a robot, regardless of its need. Thus, by including one or several control conditions, in which the robot is shown in the same situation but without indicating a need for help, future studies could address the robustness of the effect found by Martin et al. (2020). For example, it is plausible to assume that had we included no-need control conditions in the current study, helping rates in these conditions would have dropped relative to the in-need conditions regardless of the robots assigned degree of animate autonomy and friendly expressiveness. This would suggest that the overall lower rates of helping in the current study relative to Martin et al. (2020) could be due to a cohort effect.

Some support for this explanation stems from the interview data. Although the animacy scores of the five entities followed the same pattern as in Martin et al. (2020), children in the current study attributed significantly less animacy to all entities. Importantly, the current data did not provide evidence for experimenter effects, nor for material, procedural, and demographic differences, further supporting the possibility of a cohort difference across the two studies. Notably, more participants reported owning a robot in the current study than in the study by Martin et al. (2020), however, the difference in robot ownership was not significant. Moreover, while increased robot ownership could conceivably influence children’s perception of and behavior toward robots, the question remains as to why children were less likely to attribute animacy to the remaining entities in the current study.

Future studies should also aim to minimize the opportunity for cognitive conflict, especially under conditions of low autonomy. When employing a similar procedure as used in the current study, future studies should pay close attention to the procedure. Importantly, in a test phase using a robot with low autonomy, children’s target behavior (e.g., returning the target object) might not be primarily motivated by a desire to help the robot. For instance, when a second actor (e.g., the experimenter) would control the robot during the test phase, returning the object to the robot could be considered as a means to help the experimenter. In contrast, when the participant would ostensibly control the robot during the test phase, cognitive conflict might still arise when the robot drops the object unexpectedly. Moreover, returning the object in this case might be primarily driven by an egoistic desire to continue interacting with the robot.

Lastly, the results have interesting implications for the field of child-robot interaction. While the study by Martin et al. (2020) showed that young children appeared to accept a NAO robot as an agent similar to a human, it had been unclear which robot characteristics contributed to this acceptance. Although many robot features could potentially affect children’s behavior and perception, the current study can be considered a first step toward discerning the effects of certain robot characteristics on children’s behavior. Interestingly, the current results indicate that certain robot characteristics that have been shown to influence adults have little or no effect on young children. In this regard, robot engineering may face different challenges when developing robots for children and adults. For instance, a well-known challenge for robot engineering is the so-called uncanny valley effect (Mori, 1970/2012). That is, adults prefer lifelikeness in robots to a certain point, but when robots become too lifelike, adults react with unease (e.g., Ho et al., 2008; Gray and Wegner, 2012). This shift might be a result of cognitive conflict when animate and inanimate features are combined and a mismatch of expectation and perception occur (e.g., Cheetham et al., 2011; Weis and Wiese, 2017). Although research with children on the uncanny valley effect is still limited (Lewkowicz and Ghazanfar, 2011; Brink et al., 2017; Feng et al., 2018), findings by Brink et al. (2017) suggest that the uncanny valley effect emerges at around 9 years of age. Specifically, whereas younger children’s rating of the uncanniness of machinelike and realistic humanlike robots did not differ, older children rated a humanlike robot as more uncanny. Thus, taken together with the results by Brink et al. (2017), the findings of the current study further contribute to evidence suggesting that young children’s perceptions of robots may not follow the same principles as older children’s and adults’ perceptions.

This possibility raises important ethical questions, especially because child-robot interactions, including interactions without adult supervision, are expected to increase in the future (e.g., Breazeal et al., 2008; Pachidis et al., 2018). In addition to concerns regarding privacy (e.g., many robots are equipped with cameras and internet connections) and legal accountability, this trend also raises concerns regarding moral responsibilities (e.g., deception) and children’s psychological development (e.g., Tanaka and Kimura, 2009; Sharkey and Sharkey, 2010; also see Martin et al., 2020). Notably, if and how regular contact with robots would affect children’s development of the animate–inanimate distinction is currently unclear. It is possible that by increasing children’s contact with living/non-living hybrids children’s developing animate–inanimate distinction might be hindered rather than supported (Martin et al., 2020). A further issue arises by developing “personified robots that allow themselves to be treated as objects” (Kahn et al., 2012, p. 313). Because it is currently unknown if and what children learn about animacy from interactions with robots, it is also unclear whether children would extend this knowledge to their interactions with animate beings. Overall, the complexity of potential ethical issues associated with child-robot interaction highlights the need to apply psychological approaches to this field.

## Conclusion

In conclusion, the current study was designed as a first step toward discerning the effects of certain robot characteristics on children’s behavior and perception. The results provide no evidence that 3-year-old children’s instrumental helping behavior is affected by the robot recipient’s level of animate autonomy and friendly expressiveness. These findings support the idea that young children’s helping behavior is relatively indiscriminate across aspects of the recipient. Results showing that children perceived the robot as animate, regardless of its animate autonomy and friendly expressiveness, indicate that children’s helping behavior in all conditions was at least partially driven by their perception of the robot as an animate being. While the current study focused on the robot’s animate autonomy and friendly expressiveness, future work is needed to extend this approach to other robot characteristics.

## Data Availability Statement

The raw data supporting the conclusions of this manuscript will be made available by the authors, without undue reservation, to any qualified researcher.

## Ethics Statement

The studies involving human participants were reviewed and approved by the Swinburne University Human Research Ethics Committee. Written informed consent to participate in this study was provided by the participants’ legal guardian/next of kin.

## Author Contributions

DM, MM, JK, CP, and SP designed the study. DM, MM, and GC collected and coded the data. DM, JK, MM, and CP analyzed the data. DM, JK, and CP wrote the manuscript. All authors reviewed the manuscript.

## Conflict of Interest

The authors declare that the research was conducted in the absence of any commercial or financial relationships that could be construed as a potential conflict of interest.
