# Supplementary material for: Young Children’s Indiscriminate Helping Behavior Toward a Humanoid Robot
Source: Front Psychol. 2020 Feb 21;11:239. doi: 10.3389/fpsyg.2020.00239 (PMC7047927; doi:10.3389/fpsyg.2020.00239)
Supplement: Supplementary file 1 [file Presentation_1.zip › Supplementary material/Supplementary Tables.docx]

Supplementary Material

| Table S1  *Warm-up Phase Scripts* |  |
| --- | --- |
| High Autonomy | Low Autonomy |
| E1: “Would you like to ask the robot what its name is?”  Robot: “Hello! My name is Kira. Welcome to the Babylab!”  Robot: “What’s your name?”  Robot: “Nice to meet you.” | E1: “This robot has a number. Press this button and it will tell us.”  Robot: “My ID number is 19469233“  E1: “Press the button and the robot will ask you a question.”  Robot: “What’s your name?” |
| Robot: “How old are you?”  Robot: “Great! I am the same age as you.”  Robot: “I would love to play with you today!” | E1: “If you press the button the robot will ask another question.”  Robot: “How old are you?”  E1: The robot was made in a robot factory. Do you want to know how old it is? Press the button.  Robot: “I was made three years ago" |
| E1: “Would you like to ask Kira what her favourite colour is?”  Robot: “My favourite colour is blue.”  Robot: “What is your favourite colour?” | E1: “This robot can say different colours. Do you want to press the button? It will tell us what colour this is.” (E1 holds up a blue sheet).  Robot: “Blue.“  E1: “Press the button and the robot will ask another question.”  Robot: “What is your favourite colour?” |
| E1: “Would you like to ask Kira what her favourite food is?”  Robot: “My favourite food is ice cream.”  Robot: “What is your favourite food?“ | E1: “When you press the button, the robot will tell us what it eats.”  Robot: "I don't eat food, I get my energy by being plugged into the wall.”  E1: “The robot will ask you a question when you press the button.”  Robot: “What is your favourite food?” |
| E1: “Would you like to play a game with Kira? This game is about guessing animal sounds. Kira will make sounds.”  Robot plays three to four sounds, one at a time  Robot responds with “yes” or “no” to each answer | E1: “Would you like to play a game with the robot? This game is about guessing animal sounds. You can press the button and the robot will make sounds.”  Robot plays three to four sounds, one at a time  E1 (after each answer): “Press the button to find out if you were right.”  Robot: “yes” or ”no” |
| E1: “I think Kira likes music.”  Robot: “I love music!”  Robot: “Do you like music?”  Robot: “This is my favourite song!” (sings for 30s) | E1: “The robot can play music. If you press the button it will ask a question.”  Robot: “Do you like music?“  E1: “Press the button and the robot will sing.”  Robot: (sings for 30s) |
| E1: “Kira can also play with a rattle.”  Robot stretches out arm, E1 hands rattle to robot)  Robot: plays rattle for 8s (repeated if participant agreed) | E1: “The robot can also play with a rattle. Press the button.”  Robot stretches out arm E1 hands rattle to robot  E1: “Press the button to make it play.”  Robot: plays rattle for 8s (repeated if participant agreed) |
| E1: “The robot can also play a xylophone. Kira, would you like to show us?”  Robot: “Yes, I will show you. Could you lift me on the table?”  E1: “Yes, can you squat so I can lift you up?”  Robot squats  E1 sets up xylophone  E1: “I will make sure that it works.”  E1 plays xylophone for approximately 3s  E1 lifts robot onto table  E1: “Here is the stick.”  E1 hands stick to the robot  E1: “I have some work to do, but Kira will play the xylophone. You can watch her.”  E1 sits in chair, turned away from participant and robot, face covered with a folder  Robot: “I will play the xylophone now.” | E1: “The robot can also play a xylophone.”  E1: “I will make the robot squat.”  E1 presses button  Robot squats  E1 sets up xylophone  E1: “I will make sure that it works.”  E1 plays xylophone for approximately 3s  E1 lifts robot onto table  E1: “I will make the robot take the stick.” E1 presses button and hands stick to the robot  E1: “I will press the button to make the robot play the xylophone and then I will put away the robot controller because we don’t need it now.”  E1 presses button  E1: “I put the robot controller away now.”  E1 puts tablet device on top shelf of a cabinet  E1: “I have some work to do, but the robot will play the xylophone. You can watch it.”  E1 sits in chair, turned away from participant and robot, face covered with a folder |

Table S2

*Percentage of Children Helping as well as Means and Standard Deviations of Latency to Help in all Conditions of the Current Study and the Study by Martin et al. (2020)*

|  |  | Latency | |
| --- | --- | --- | --- |
|  | Percentage of Help | Mean (in sec) | Standard Deviation |
| HAAF | 45 | 7.65 | 5.97 |
| HAAN | 65 | 6.69 | 3.29 |
| LAAF | 38 | 7.51 | 3.91 |
| LAAN | 48 | 6.88 | 4.27 |
| Experimental Condition | 70 | 6.76 | 2.76 |
| Control Condition | 30 | 17.45 | 8.23 |
